# Cognitive Impairment in Heart Failure—A Review

**DOI:** 10.3390/biology11020179

**Published:** 2022-01-23

**Authors:** Fang Qin Goh, William K. F. Kong, Raymond C. C. Wong, Yao Feng Chong, Nicholas W. S. Chew, Tiong-Cheng Yeo, Vijay Kumar Sharma, Kian Keong Poh, Ching-Hui Sia

**Affiliations:** 1 Department of Cardiology, National University Heart Centre Singapore, Singapore 119074, Singapore; fangqin.goh@mohh.com.sg (F.Q.G.); william_kong@nuhs.edu.sg (W.K.F.K.); raymond_cc_wong@nuhs.edu.sg (R.C.C.W.); nicholas_ws_chew@nuhs.edu.sg (N.W.S.C.); tiong_cheng_yeo@nuhs.edu.sg (T.-C.Y.); 2 Department of Medicine, Yong Loo Lin School of Medicine, National University of Singapore, Singapore 119074, Singapore; vijay_kumar_sharma@nuhs.edu.sg; 3 Division of Neurology, Department of Medicine, National University Hospital, Singapore 119074, Singapore; yao_feng_chong@nuhs.edu.sg

**Keywords:** heart failure, cognitive impairment, dementia, cerebral haemodynamics

## Abstract

**Simple Summary:**

Compared to the general population, patients with heart failure have reduced cognition and increased dementia risk. Brain changes have been observed in these individuals, including reduced brain volumes and abnormal areas suggestive of ischaemia (lack of blood and hence oxygen supply to tissues). Patients with heart failure who have cognitive impairment have poorer self-care and are at increased risk of rehospitalisation and death. Causes of cognitive impairment in heart failure have been suggested, including reduced blood supply to the brain, inflammatory processes, protein abnormalities and thromboembolic disease (formation of blood clots which may travel to the brain and impede blood flow). In this article, we discuss these potential causes linking heart failure and cognitive impairment, and discuss the recognition and management of cognitive impairment in patients with heart failure.

**Abstract:**

Cognitive impairment (CI) is common in heart failure (HF). Patients with HF demonstrate reduced global cognition as well as deficits in multiple cognitive domains compared to controls. Degree of CI may be related to HF severity. HF has also been associated with an increased risk of dementia. Anatomical brain changes have been observed in patients with HF, including grey matter atrophy and increased white matter lesions. Patients with HF and CI have poorer functional independence and self-care, more frequent rehospitalisations as well as increased mortality. Pathophysiological pathways linking HF and CI have been proposed, including cerebral hypoperfusion and impaired cerebrovascular autoregulation, systemic inflammation, proteotoxicity and thromboembolic disease. However, these mechanisms are poorly understood. We conducted a search on MEDLINE, Embase and Scopus for original research exploring the connection between HF and CI. We then reviewed the relevant literature and discuss the associations between HF and CI, the patterns of brain injury in HF and their potential mechanisms, as well as the recognition and management of CI in patients with HF.

## 1. Introduction

**Figure 1:**
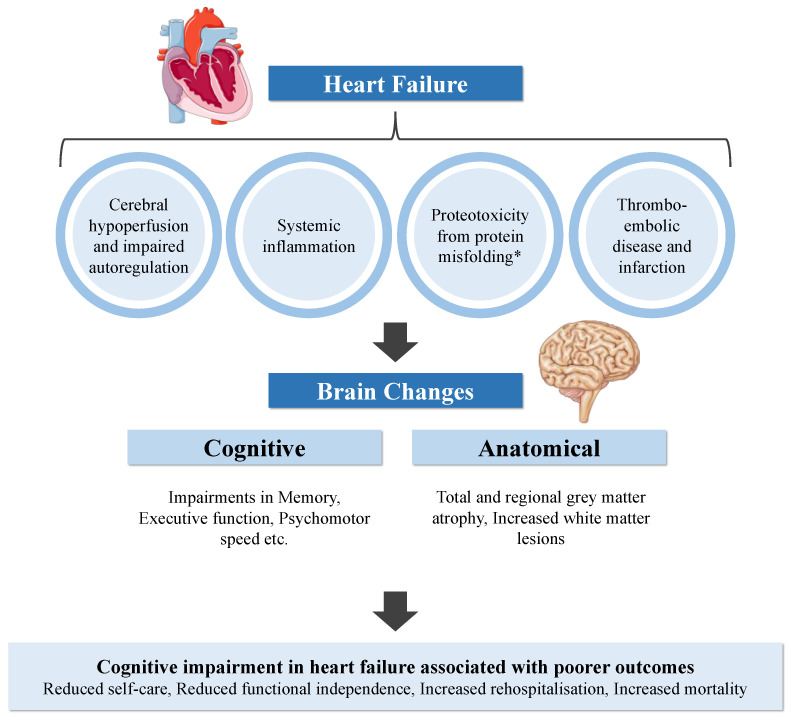
Summary of the reported pathophysiology, brain changes and impact of cognitive impairment in heart failure. *Proteotoxicity may be a shared disease pathology between specific cardiomyopathies and CI.

Cognitive impairment (CI) in patients with heart failure (HF) is common, with a reported prevalence of 20–80% [1,2,3,4,5,6,7,8]. Patients with HF demonstrate increased cognitive deficits compared to controls in several cognitive domains [9] and have poorer self-care and treatment adherence [10]. Cerebrovascular haemodynamics and structural brain changes have been postulated to contribute to the cognitive deficits seen in patients with HF [11]. More importantly, CI in HF is associated with a poor prognosis [12]. Although CI is prevalent among patients with HF and has a significant impact on these individuals [12,13], the pathophysiology behind how HF influences cognitive function remains poorly understood. In this article, we review the associations between HF and CI, the patterns of brain injury in HF and their potential mechanisms, as well as the recognition and management of CI in patients with HF. A graphic summary of the reported pathophysiology, brain changes and impact of CI in HF is shown in Figure 1.

## 2. Methods

We performed a search on MEDLINE, Embase and Scopus on 1 December 2021, for articles from inception until 1 December 2021, and included the following terms: (cognition OR confusion OR cognitive deficit OR cognitive decline OR cognitive impairment OR dementia OR Alzheimer* OR neuropsych* test OR neuropsych* deficit OR memory OR neuroimaging) AND (heart failure OR cardiac failure OR reduced ejection fraction OR myocardial dysfunction OR systolic dysfunction OR diastolic dysfunction) and other related terms. The included literature comprised original research involving humans, published in a peer-reviewed journal. Studies exploring the (1) associations between HF and CI, (2) brain changes in HF, (3) proposed mechanisms behind how HF may contribute to CI, (4) impact of CI in HF and (5) effect of HF therapies on CI were included. The search was not restricted by language of publication. Case series and case reports were excluded. Titles and abstracts were screened and additional articles were identified from handsearching the references of reviews. A full text review was performed for all relevant articles.

## 3. Epidemiology of HF and Prevalence of CI

An estimated 64.3 million people are living with a diagnosis of HF worldwide, with an increasing prevalence due to population ageing and improved survival after diagnosis. While HF is primarily a disease of older age, the number of younger individuals with HF appears to be on the rise [14]. This may be due to an increase in the prevalence of obesity and its related comorbidities such as type 2 diabetes mellitus, hypertension and atrial fibrillation [15]. Some have also suggested that the improved survival of patients with congenital heart disease may contribute to this increase, although this has not been specifically studied [14]. CI was previously thought to be limited to older patients with HF, but has since been described in younger HF populations as well [16,17]. While the prevalence of CI in HF has been reported in many studies, there is significant heterogeneity in the existing literature [9,17,18]. Patient populations are diverse, with some studies including patients with stable, chronic HF [19,20] and others including those with acute decompensated HF [3,5,8]. Sterling and Hammond et al. specifically studied incident HF [21,22]. There is also a lack of standardisation of the cognitive assessment tools used. For example, screening tests such as the Mini Mental State Examination (MMSE) [6], Montreal Cognitive Assessment (MoCA) [7,23] and Hodkinson Abbreviated Mental Test (AMT) [3,13] were used by some investigators to assess CI, while others used more comprehensive neuropsychological batteries [2,19,24,25]. Recognising these limitations, CI at least appears to be common in HF and is present across a wide age range.

## 4. Cognitive Changes in HF

Compared to healthy controls, patients with HF demonstrate reduced global cognition as well as deficits in multiple cognitive domains including executive function, psychomotor speed and verbal memory [9]. Sterling et al. found that the prevalence of CI among patients with incident HF (14.9%) was similar to controls without HF (13.4%) and was lower than reported in the general HF population. This suggests that CI may develop at some point after the onset for HF, rather than it being present prior to HF diagnosis or due to concomitant cardiovascular risk factors [21]. This is further supported by a study by Hammond et al. which reported a greater decline in Modified Mini Mental State test scores of 10.2 points over 5 years in patients with incident HF, compared to 5.8 points in controls [22].

HF has also been associated with an increased risk of dementia that may not be limited to vascular dementia [26,27]. Adelborg et al. found that patients with HF were 1.5 times more at risk of developing vascular dementia, and were also 1.3 times more likely to develop other dementias (defined as any dementia apart from vascular dementia or Alzheimer’s disease) over a 35-year follow-up period. However, they did not find a difference in the risk of Alzheimer’s disease between HF patients and controls [27]. In contrast, Qiu et al. reported an increased risk of both all-cause dementia and Alzheimer’s disease in patients with HF within a community-based cohort. Over a 9-year follow-up period, patients with HF were approximately 1.8 times more likely to develop incident all-cause dementia and 1.8 times more likely to develop Alzheimer’s disease [28]. Compared to the Adelborg study which identified incident dementia and Alzheimer’s disease from a psychiatric registry [27], Qiu et al. evaluated their study population on three separate follow-up sessions with a comprehensive clinical examination and cognitive test battery, with corroboration between two independent physicians. Therefore, potential misidentification of dementia and misclassification of dementia subtype in the Adelborg study may have contributed to these discrepancies in results [27]. In the general population, Jefferson et al. observed that a lower cardiac index (defined as cardiac output divided by body surface area measured in L/min/m^2^) among subjects of the Framingham Offspring Cohort was associated with higher all-cause dementia and Alzheimer’s risk [29].

### 4.1. HF Severity and Degree of CI

A dose–response relationship between HF and CI would further lend support to a connection between the two diseases. Patients with a more advanced New York Heart Association (NYHA) class demonstrated lower overall Z-scores compared to those with NYHA I or II disease and increased HF severity was associated with reduced memory, visuospatial ability, psychomotor speed and executive function [20]. Harkness et al. also found that the incidence of CI, defined as MoCA score < 26, was greater in HF patients with NYHA III or IV class (91%) compared to NYHA I or II class (52%) [23]. Hanon et al. reported more severe memory impairment, as evaluated by the delayed-recall Memory Impairment Screen (MIS-D), in patients with higher NYHA class [30] and Lee et al. found that NYHA class II or higher was independently associated with an increased likelihood of cognitive decline in patients with HF [31]. Similarly, another study reported poorer attention and memory in HF patients who scored higher on dyspnoea and fatigue rating scales [32]. Kindermann et al. observed poorer cognition in patients with decompensated HF compared to those with stable HF, and found that cognition improved after HF compensation [25]. In contrast, Huijts et al. found that although severe CI was present at baseline more often in HF patients with NYHA IV compared to NYHA II class, the prevalence of severe CI remained stable over 18 months in both groups. Moreover, baseline HF severity was not associated with cognitive decline [13]. These differing findings may be due to the authors’ use of the AMT to determine CI, which may be more susceptible to ceiling effects compared to other tools [33]. Myocardial stretch stimulates the release of pro B-type natriuretic peptide (proBNP), which is then rapidly cleaved into biologically active C-terminal BNP and inert N-terminal proBNP (NT-proBNP) [34]. BNP and NT-proBNP are both indicators of HF severity [35]. A connection between higher BNP levels and poorer attention and executive function was previously reported [36], in addition to reduced hippocampal volume in patients with higher BNP [37]. NT-proBNP has also been associated with an increased risk of dementia in an elderly community-dwelling population [38]. Overall, these studies suggest that HF severity may have an impact on the level of CI but the exact relationship remains to be elucidated.

### 4.2. The Impact of Ejection Fraction on CI

Left ventricular ejection fraction (EF), defined as a percentage of stroke volume over end-diastolic volume (SV/EDV ×100%), is the central measure of left ventricular systolic function. A lower left ventricular ejection fraction (EF), especially when <30%, has been associated with lower cognitive scores [39,40]. A study by Festa et al. showed that in patients 63 years old or older, EF < 30% was associated with poorer memory whereas memory was stable across all EF levels in younger patients [41]. It is unclear if this is due to poorer compensatory capacity, since age was not shown to affect dynamic cerebrovascular autoregulation in a healthy population [42]. Elderly patients do, however, appear to be more susceptible to watershed infarcts from cerebral hypoperfusion [43]. In contrast, a similar rate of cognitive decline was found in patients with HF with reduced (HFrEF) and preserved EF (HFpEF) [22] despite different patterns of cognitive deficits depending on predominance of systolic or diastolic dysfunction [36,44,45]. Concomitant severe systolic and diastolic dysfunction may worsen CI, especially in the form of poorer verbal fluency compared to those with systolic dysfunction alone [40].

### 4.3. Potential Confounders in the Association between HF and CI

HF and CI share several risk factors and studies which include a control group of patients with cardiovascular disease without HF may be useful to reduce the effects of potential confounding factors. Studies have shown that CI remains more common in patients with HF even when compared to these cardiac controls [2,24]. Vogels et al. reported that 25% of patients with HF had CI compared to 15% of those with cardiovascular disease without HF [2]. Another study found that the prevalence of abnormal performance on at least 3/7 tests in a neuropsychological battery was 57.9% and 43% in patients with severe and moderate HF, respectively, as compared to 34.3% in those with other cardiovascular diseases [24]. The degree of CI also appears to be greater in patients with HF, and HF and IHD patients were found to have a Cambridge Cognition Examination (CAMCOG) score of 2.8 and 1.8 less than healthy controls, respectively [16]. In contrast, a prospective study over 2 years found that while cognitive decline was greater in HF patients than in healthy controls, it was similar to those with coronary artery disease [46].

## 5. Anatomical Brain Changes

### 5.1. Grey Matter Atrophy

Cerebral grey matter (GM) atrophy is a feature of normal ageing. The distribution of age-related GM atrophy is not homogenous and predominantly affects the frontal, insular and cingulate cortices [47]. In general, increased GM atrophy has been observed in patients with HF. Almeida et al. found that patients with HF demonstrated more extensive cortical and subcortical GM losses especially in the subcortical nuclei, caudate, anterior cingulate and frontal lobes, which are important regions for demanding cognitive activity such as attention and memory [16]. In a follow-on study, the authors did not find a significant decrease in total GM volume and cognitive function after 2 years in those with HF, although subtle regional GM losses were observed [48]. In both studies, GM changes in patients with HF were more pronounced than in patients with IHD without HF when compared to healthy participants. This suggests that while concomitant cardiovascular disease may contribute to GM loss, it does not fully explain the degree of GM atrophy seen in HF.

Increased medial temporal lobe atrophy (MTA) has been demonstrated in patients with HF. The medial temporal lobe includes the hippocampus, amygdala and parahippocampal regions and is mainly involved in the encoding, storage and retrieval of episodic and spatial memory [49]. Frey et al. found that patients with HF had an approximately 11-fold greater risk of MTA and demonstrated deficits in attention and memory corresponding to the degree of MTA. However, progressive hippocampal volume loss over 3 years was no different from that of physiological ageing [19,50]. Notably, this study consisted of patients with non-progressive HF. Throughout the 3-year observation period, NYHA II remained the most frequent class, with no change in left ventricular EF or 6-minute walking distance (a test of aerobic capacity and endurance). Cognitive function also remained stable in these patients [50]. This suggests that brain injury and CI may not worsen significantly in patients with stable disease [51]. Apart from attention and memory, MTA has also been associated with poorer executive function in patients with HF [52]. Regional GM loss is also seen in other brain structures in HF patients, including the putamen, mammillary bodies and areas of the cortex corresponding to autonomic function, cognitive function, affect, language and vision [53,54].

### 5.2. White Matter Lesions

White matter lesions (WMLs) are seen in normal ageing and are more prevalent in the elderly. However, they are also known to be associated with cerebrovascular risk factors and cerebral ischaemia. Although WM hyperintensities may be detected on T2-weighted magnetic resonance imaging (MRI) in asymptomatic individuals, they have also been associated with CI [55,56]. Vogels et al. found that patients with HF had more WMLs and lacunar infarcts compared to both healthy participants and patients with other cardiovascular diseases [55]. In addition, a recent study by Stegmann et al. demonstrated an increase in WMLs with a longer HF disease duration [57]. Frey et al., however, found that the extent of WMLs in patients with HF was not increased at baseline and progressed within the limits of physiological ageing [19,50]. This may be attributed to the large proportion of stable HF patients in this study [50].

## 6. Proposed Aetiologies of CI in HF

**Figure 2:**
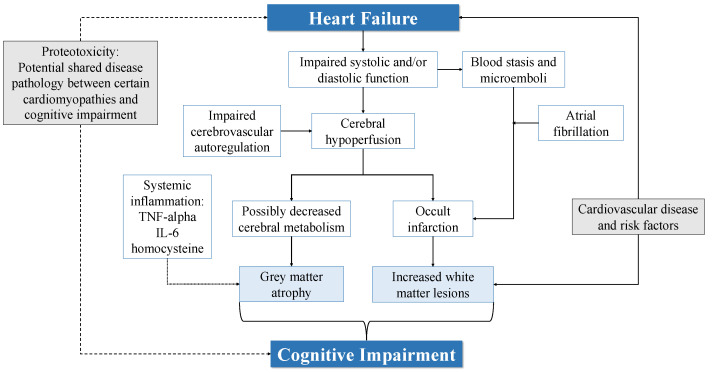
Summary of potential pathophysiological pathways linking heart failure and cognitive impairment. Dotted line: the mechanisms by which systemic inflammation may contribute to brain changes and cognitive impairment in heart failure are not well described. Dashed line: proteotoxicity may be a shared disease pathology between specific cardiomyopathies and CI. IL, interleukin; TNF, tumour necrosis factor.

Several pathophysiological pathways have been proposed to contribute to the structural brain changes and CI among patients with HF. These are outlined in Figure 2.

### 6.1. Cerebral Hypoperfusion and Impaired Autoregulation

Reduced cerebral blood flow (CBF) is one of the proposed mechanisms of brain injury and CI in HF [55,58]. Several studies have demonstrated lower CBF velocities on transcranial Doppler (TCD) in those with HF [59,60,61]. Furthermore, TCD measurements in patients with HF showed a decline over a 12-month period [62]. Lower CBF in HF patients impacted global cognitive function, attention and executive function [62,63], while reduced regional hippocampal CBF was associated with poorer performance on measures of delayed memory [64]. A low output state in HF may result in chronic cerebral hypoperfusion in patients with HF, making them more susceptible to watershed infarcts. Additionally, owing to the similar risk factors shared by patients with HF and cerebrovascular disease, patients with HF may also have a poorer collateral blood supply due to atheromatous stenosis of the cerebral arteries [43]. Some studies compared CBF measurements against structural neuroimaging or neuropsychological testing and evaluated the relationship between CBF and CI in HF [55,59]. Alosco et al. found that reduced CBF in patients with HF was associated with increased WMLs, which were in turn related to poorer MMSE scores [59]. Vogels et al. similarly described lower CBF in patients with HF, but did not find a correlation with brain changes on neuroimaging [55].

In the general population, Jefferson et al. reported higher MRI-assessed cardiac index to be positively related to total brain volume and information processing speed [58]. Similarly, a lower cardiac index was associated with increased dementia risk [29]. In a study of 4366 individuals from the United Kingdom Biobank, van Hout et al. found that individuals with subclinical reduced left ventricular EF had reduced total brain volume and GM volume, and also increased WMLs [65]. Interestingly, only WM and hippocampal volume loss were associated with CI, and both were not related to EF [65]. Arterial stiffness, microvascular damage, atherosclerosis and inflammation in HF may possibly confound the reported relationship between EF and CI. These pathophysiological mechanisms are adversely associated with both cardiac function and cognition, making it difficult to ascertain the true association between EF and CI [66,67]. However, Park et al. found that total brain volume and hippocampal volume remained associated with poorer left ventricular systolic function even after adjustment for cardiometabolic disease [37]. Left ventricular stroke volume and cardiac output have also been linked to CI [68]. The potential mechanisms by which cardiac dysfunction may influence brain atrophy are not well understood but may be related to decreased cerebral metabolism. Patients with HF with extensive hibernating myocardium had reduced cerebral metabolism in frontal and hippocampal areas in a study utilising 18F-flurodeoxyglucose positron emission tomography/computed tomography (18F-FDG PET/CT) imaging [69].

A reduced CBF is unlikely to be the sole explanation for cortical GM loss [16,54]. Brain areas such as the periventricular white matter, basal ganglia and hippocampus are susceptible to cerebral hypoperfusion due to their location at the junction of large-vessel arterial territories, or due to their irrigation by long-penetrating end arterioles. In contrast, the cortex has a rich dual blood supply and can better tolerate cerebral hypoperfusion [43,70]. The interplay of other cardiovascular risk factors may also contribute to cortical GM loss, since similar patterns of GM loss have been observed in HF and IHD patients [16]. Leeuwis et al. further argued that CBF may not be the main reason for CI in HF in light of their findings that while CBF was lower in patients with HF, this did not correspond with reduced cognitive function [71].

CBF has been shown to increase after heart transplantation [72] and heart transplantation has been associated with improved cognitive function [73,74]. Cognitive improvement has also been reported after left ventricular assist device (LVAD) placement [75,76]. However, these improvements were marginal and MoCA scores increased by approximately 1.6 following LVAD placement [76]. Schall et al., on the other hand, did not find a significant difference between pre- and post-operative cognitive scores after 7.7 months in their patients with dilated cardiomyopathy who underwent heart transplantation, despite greatly improved physical health [77]. A possible explanation is the shorter follow-up duration compared to other studies [73]. Several cognitive scores showed a non-statistically significant increase and a longer reassessment interval may have revealed further cognitive improvement [77]. Another reason may be the use of an extensive neuropsychological battery by Schall et al. compared to less rigorous screening measures such as the MoCA in other studies [74]. Patients with HF may also have diminished cerebrovascular autoregulation, with greater impairments in those with NYHA IV compared to NYHA II and III. Accordingly, cerebral oxygen saturations were found to be lower in patients with HF [78,79]. Previous studies have shown a blunted haemodynamic response and greater CBF reduction in patients with HF in response to upright posture [80,81]. More recently, Kharraziha et al. observed a more pronounced decrease in cerebral tissue oxygen saturations in response to head-up tilt in patients with HF [82]. While it is unclear how HF may lead to impaired autoregulation, it could result in increased susceptibility to low cardiac output states due to an inability to maintain CBF via vasodilatory mechanisms.

### 6.2. Systemic Inflammation

The systemic inflammatory state recognised in patients with HF may further contribute to CI in HF. Tumour necrosis factor (TNF)-alpha, interleukin (IL)-6 and cortisol are markers of inflammation which, together with high total plasma homocysteine (tHcy), have been associated with neuronal degeneration [83]. Increased secretion of cytokines was previously shown to correlate with decreased memory performance [84]. Patients with HF may demonstrate enhanced expression and release of inflammatory cytokines, with elevated levels of circulating cytokines proportionate to NYHA class and cardiac performance [85,86]. High tHcy was shown in a study by Almeida et al. to be independently associated with cerebral GM loss in HF [16].

### 6.3. Proteotoxicity

The possibility of proteotoxicity contributing to the development of both HF and CI has also been explored [87]. Misfolded proteins aggregate to form soluble oligomers, soluble aggregates and finally associate to form inclusion bodies. These aggregated proteins may induce cell death and this process is known as proteotoxicity. Misfolded proteins are associated with neurodegenerative diseases such as Alzheimer’s disease, Huntington’s disease and Parkinson’s disease [87,88]. Protein misfolding has also been implicated in certain cardiomyopathies. One such example is cardiac amyloidosis, where misfolded monocloncal immunoglobulin light chains or transthyretin results in the aggregation of amyloid fibrils. Extracellular deposition of these proteins in the myocardium results in myocardial distortion [89]. Upregulation of cytoskeletal, linkage and extracellular proteins have also been found in dilated cardiomyopathy [90]. Although some have suggested that protein misfolding may represent a shared pathophysiology between HF and neurodegenerative diseases [91], these specific cardiomyopathies are relatively uncommon causes of HF and hence proteotoxicity is unlikely to be a shared aetiological factor for HF and CI in the vast majority of patients with HF.

### 6.4. Thromboembolic Disease and Cerebral Infarction

There is considerable evidence in the literature linking atrial fibrillation (AF) and risk of CI and dementia [92]. Patients with HF and concomitant AF were shown to have worse global cognition and memory as well as reduced CBF velocities [93]. The association between AF and CI in those without clinical stroke suggests that occult embolic disease may contribute to cognitive decline in these patients [92]. While the impact of AF on CI is compelling [92], it cannot fully explain CI in HF as CI remains prevalent in patients with HF after controlling for AF [2]. In those with HF in sinus rhythm, downregulation of thrombomodulin, reduced myocardial contractility and resultant stasis of blood in HF may also lead to microemboli and occult cerebral infarction [94]. Hypercoagulability and increased risk of venous thromboembolism in HF further increase thromboembolic risk [95].

## 7. Impact of CI on Prognosis in HF

Although the mechanisms proposed for CI in HF are multifactorial and incompletely understood, the negative impact of CI on patients with HF are well known. Deficits in executive function in patients with HF have been associated with poor functional independence, decreased ability to manage medications as well as non-compliance to smoking cessation [10]. Functional decline in these patients has also been demonstrated in prospective studies [96]. Cognitively impaired HF patients also tend to display lower levels of self-care and self-confidence [97]. Reduced medication adherence in patients with HF [98] is particularly concerning as these patients tend to be on several medications, many of which confer significant mortality and morbidity benefit. Inability to self-care is also likely to negatively impact fluid and dietary restrictions as well as recognition of symptoms of decompensation. It is therefore unsurprising that CI is associated with increased rehospitalisations in these patients [12,99]. Short- and longer-term mortality is also higher in HF patients with severe and milder CI [13,99,100] although worst outcomes are seen in those with severe cognitive dysfunction [101]. The relationship between CI and mortality is not limited to sicker patients in hospital [12] and has also been observed in stable outpatient HF populations [102].

## 8. Screening for CI in Patients with HF

Despite the prognostic implications and management considerations of CI in HF, CI remains poorly recognised by physicians [6] as well as cardiologists [30]. A complete neuropsychological battery is unlikely to be practical for all patients but brief screening tests for CI may be valuable [103]. A systematic review by Cameron et al. concluded that the MMSE had a low sensitivity (26%) but high specificity (95%) [104], and others suggested that the MoCA may be a better screening tool [103]. Hawkins et al. compared the MoCA and MMSE against a gold standard neuropsychological test battery in patients with HF and found that both MoCA score < 25 and MMSE score < 28 were optimally sensitive and specific (MoCA: 64% sensitive, 66% specific; MMSE 70% sensitive, 66% specific) [105]. The Mini-Cog is a quicker test and may be useful in dementia but is limited in mild CI [106]. More comprehensive testing in selected high-risk patients may be useful, and patients with HF performed especially poorly in the Trail Making Test B, Symbol Digit Modality Test and California Verbal Learning Test compared to controls [9]. However, there are currently no guidelines on screening for CI in HF.

## 9. Impact of HF Therapies on CI

Angiotensin converting enzyme (ACE) inhibitor use has been associated with improved cognition in patients with HF independent of blood pressure changes. The degree of cognitive improvement appears to be greater with higher ACE inhibitor dose and treatment duration [107]. ACE inhibitors may be involved in cerebrovascular autoregulation and have also been suggested to modulate neuronal regeneration processes via angiotensin II type 2 (AT2) receptors following neuronal injury [108]. CRT improves cardiac function and reduces morbidity and mortality in selected HF patients [109]. CRT implantation has also been associated with increased CBF [110] as well as cognitive improvement in patients with HF [111,112]. Fumagalli et al. found that patients with HF on optimal medical therapy who then underwent CRT implantation had improved cognitive function 6 months after the procedure, although the effect was small (increase in MMSE score by 1.1 points) [111]. Biventricular defibrillators (CRT-Ds) were also shown to be associated with significant cognitive improvement compared to implantable cardioverter defibrillators (ICDs) in controls, in terms of concentration ability and scores of global cognition [112]. Similar to heart transplantation [73,74] and LVAD placement [75,76], the delay or reversal of CI in patients with HF following CRT implantation may be due to enhanced cardiac function. In addition, functional performance measured by the Short Physical Performance Battery (SPPB) has been shown to improve following CRT implantation [111]. Dedicated, supervised exercise training [113] and nurse-led management programmes [114] have also been shown to improve cognitive function in HF patients with CI. Deficits in global cognition, attention, executive function, psychomotor function and memory were reduced after cardiac rehabilitation in older adults with cardiovascular disease [115].

## 10. Future Challenges

Previous systematic reviews and meta-analyses have been limited by the heterogenous literature [17,18,116]. However, the overall evidence suggests that there is a connection between HF and CI, possibly mediated by HF-induced brain injury. Further studies incorporating measures of cardiac function, neuroimaging and comprehensive neuropsychological assessments will be useful to investigate these heart–brain interactions. At present, such studies are rare [50]. The incidence of CI in HF is also largely unknown, although some have investigated incident CI in relation to cardiological measures in the general population [29]. Prospective studies following patients with incident HF and no previous CI will be especially useful to evaluate the development of CI corresponding to disease duration.

The negative impact of CI on patients with HF and its association with morbidity and mortality highlight the importance of prompt diagnosis and management. Screening for CI in HF is rarely performed in the clinical setting and cognitive dysfunction in patients with HF remains poorly recognised [6,30]. In a study of 282 elderly patients with HF identified to have CI via the MMSE, only 22.7% had their CI documented by physicians during clinical encounters. Those who had less severe impairment were more likely to have their CI missed. Importantly, this study found that 6-month mortality or hospital readmissions were increased in patients with undocumented CI but not in those with documented CI, when compared to patients with normal cognition [6]. Similarly, Hanon et al. studied 912 ambulatory patients with HF and found that 45.6% had memory impairment identified via the MIS-D, whereas cardiologists only suspected memory impairment in 12% before the test [30]. Administration of a global cognitive screening tool such as the MoCA or MMSE in outpatient clinics or during admissions for HF may help to identify patients with CI. Metabolic derangements and other causes of acute delirium should be excluded, especially in patients with decompensated HF. Follow-up at a nurse-led outpatient clinic was found to improve cognitive function and knowledge of self-care [114], suggesting that a trial of similar interventions as resources allow may be of use. With the potential benefits of ACE inhibitors on cognitive function, closer monitoring of medication adherence in patients with HF may be more important than ever. The increased burden of frequent follow-up clinic visits on healthcare resources can be alleviated by the use of telemedicine.

## 11. Conclusions

Current evidence supports a relationship between HF and CI. Brain injury in HF is not yet well understood and further study is needed to elucidate the underlying pathophysiological mechanisms. The prognostic implications and potential reversibility of CI in HF highlight the importance of early disease recognition.
